# Simulation of the potential distribution of rare and endangered *Satyrium* species in China under climate change

**DOI:** 10.1002/ece3.9054

**Published:** 2022-07-11

**Authors:** Xianheng Ouyang, Shihao Bai, Garry Brien Strachan, Anliang Chen

**Affiliations:** ^1^ School of Forestry and Biotechnology Zhejiang A&F University Hangzhou China; ^2^ Shanghai Center for Systems Biomedicine Shanghai Jiao Tong University Shanghai China; ^3^ School of Humanities and Law Zhejiang A&F University Hangzhou China

**Keywords:** environmental variables, MaxEnt, potential suitable habitat, *Satyrium*

## Abstract

*Satyrium* is an endangered and rare genus of plant that has various pharmacodynamic functions. In this study, optimized MaxEnt models were used in analyzing potential geographical distributions under current and future climatic conditions (the 2050s and 2070s) and dominant environmental variables influencing their geographic distribution. The results provided reference for implementation of long‐term conservation and management approaches for the species. The results showed that the area of the total suitable habitat for *Satyrium ciliatum* (*S. ciliatum*) in China is 32.51 × 10^4^ km^2^, the total suitable habitat area for *Satyrium nepalense* (*S. nepalense*) in China is 61.76 × 10^4^ km^2^, and the area of the total suitable habitat for *Satyrium yunnanense* (*S. yunnanense*) in China is 89.73 × 10^4^ km^2^ under current climatic conditions. The potential suitable habitat of *Satyrium* is mainly distributed in Southwest China. The major environmental variables influencing the geographical distribution of *S. ciliatum* were isothermality (bio3), temperature seasonality (bio4), and mean temperature of coldest quarter (bio11). Environmental variables such as isothermality (bio3), temperature seasonality (bio4), and precipitation of coldest quarter (bio19) affected the geographical distribution of *S. nepalense*; and environmental variables such as isothermality (bio3), temperature seasonality (bio4), and lower temperature of coldest month (bio6) affected the geographical distribution of *S. yunnanense*. The distribution range of *Satyrium* was extended as global warming increased, showing emissions of greenhouse gases with lower concentration (SSP1‐2.6) and higher concentration (SSP5‐8.5). According to the study, the distribution of suitable habitat will shift with a change to higher elevation areas and higher latitude areas in the future.

## Graphical abstract

Precipitation (Bio19) and temperature (Bio4, Bio3, Bio6 & Bio11) are the main environmental factors for the potential geographical distribution of Satyrium’s. The distribution range of Satyrium’s extended with global warming increased, in both situations’ emissions of greenhouse gases with lower concentration (SSP1‐2.6) and the emission of greenhouse gases with higher concentration (SSP5‐8.5).

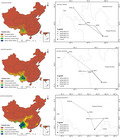

## 1 INTRODUCTION

Global warming is one of the crucial environmental problems the world faces today (Bayer et al., 2021). The global temperature has risen by about 1°C in the past century, especially in the past 30 years, causing some plant species to move to higher elevation and higher latitude areas, as the Fifth Assessment Report (AR5) of the United Nations Intergovernmental Panel on Climate Change reported (Allen et al., 2014). Climate change in the future will affect the distribution range of species, resulting in a loss of biodiversity and the extinction of endangered species (Bellard et al., 2012). Under climate change conditions, the prediction of suitable habitats of species will be considered important in the future (Kumar et al., 2021).

In research on the geographical distribution range of plants affected by climate change, the species distribution model (SDM) uses distribution data of species and environmental variables in species habitats to predict the realized niche of species (Elith & Leathwick, 2009). These data are combined with environmental data in different periods for simulating the potential distribution areas of species in those periods (Araújo & Peterson, 2012). Among the many algorithms used in modeling species distribution, the maximum entropy approach (MaxEnt) has demonstrated fast modeling, wide use, high accuracy, and stability even with small sample sizes (Merow et al., 2013; Pearson et al., 2007). So it has been the most commonly used species distribution model (Phillips et al., 2006). If the MaxEnt model and ArcGis predict potential species distributions and biodiversity risks, relative strategies can be established to reduce climate change's negative influence on global biodiversity.

*S*a*tyrium* (Orchidaceae) is a rare and endangered plant genus with about 92 species—mostly found in Africa, with five species found in Madagascar alone, and four species found in Asian countries (Mahendran & Bai, 2009). Only three species have been found in China alone (all of which are endemic in China) (Cun, 2005). They are mostly distributed throughout Southwest China. *Satyrium* is also used for traditional herbal medicinal purposes; for example, Greek medics used *Satyrium* tubers as aphrodisiacs (Teoh, 2016). Traditional health‐care centers in India use the tubers of *S*a*tyrium nepalense* (*S. nepalense*) to make energy tonics and cure different types of fevers (Mishra et al., 2018). The number of *Satyrium* resources has been rapidly diminishing. Its population has a fragmented distribution, and it is in a rare and endangered status because of the value of herbal medicine and the serious deterioration of the ecological environment in recent times (Mahendran & Bai, 2009). Changes in climate will result in changes in the biological phenology period. This in turn will result in changes in the geographical distribution of species and an acceleration in the rate of species extinction. Therefore, it is necessary to adequately understand the changing trends of the geographical distribution of species under climate change conditions and to develop relative protection strategies.

Geographical distributions of three species from *Satyrium* genus were predicted by employing the MaxEnt model: (1) In current climatic conditions, the geographical distributions of *Satyrium* species in China and the relationships between these distributions and environmental variables were studied. (2) The main environmental variables limiting their potential geographical distribution were outlined. (3) The potential geographical distribution and shifting trends of the centroid of suitable habitats in China were predicted in future climate change scenarios. This study could offer a scientific foundation for the suitable protection and use of *Satyrium*.

## 2 MATERIALS AND METHODS

### 2.1 Data of species occurrence

**FIGURE 1:**
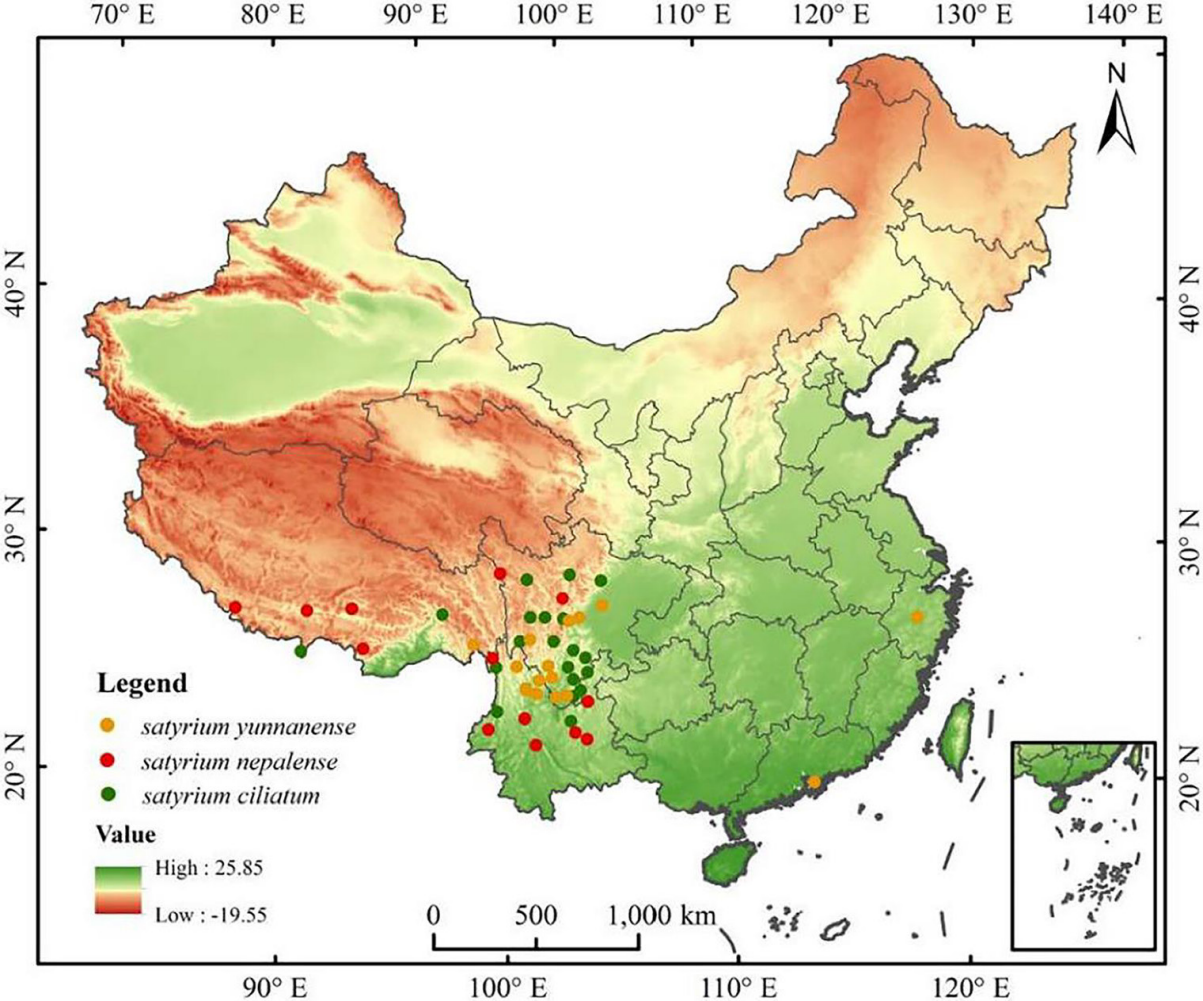
Distribution records of *Satyrium ciliatum*, *Satyrium nepalense*, and *Satyrium yunnanense* in China

Distribution records of *Satyrium ciliatum*, *Satyrium nepalense*, and *Satyrium yunnanense* in China were obtained from the National Plant Specimen Information Infrastructure (http://www.nsii.org.cn/), Chinese Virtual Herbarium (http://www.cvh.ac.cn/), and the Global Biodiversity Information Facility (https: www.gbif.org/). Geographical coordinates were not recorded in detail at some distribution points. Thus, Google Earth (http://ditu.google.cn/) was used to obtain the geographical coordinates of the points. Points with repeated latitude and longitude and missing points with latitude and longitude were deleted. A total of 44, 20, and 16 distribution records of *S. ciliatum*, *S. nepalense*, and *S. yunnanense* were gathered and used to run the MaxEnt model simulation (Figure 1).

### 2.2 Environmental variables

The World Climate Database (http://www.worldclim.org/) was used to download current (1970–2000) and future climate data (2050: 2041–2060, 2070: 2061–2080), including 19 bioclimatic variables with a resolution of 2.5′. Future climate data (2050: 2041–2060, 2070: 2061–2080) were carried out by the BCC‐CSM2‐MR climate system model, which was developed by the National Climate Center (Zhou et al., 2021). New future pathways based on socioeconomic assumptions are called shared socioeconomic pathways (SSPs), which describe the various levels of socioeconomic development used in the study (Riahi et al., 2017). The SSPs include the high‐forcing scenario (SSP5‐8.5), medium‐forcing scenario (SSP2‐4.5), and low‐forcing scenario (SSP1‐2.6). High‐ and low‐emission scenarios were represented by SSP5‐8.5 and SSP1‐2.6 in the study, respectively. Topographic parameters (altitude, slope, and aspect) were downloaded from World Climate data (http://www.worldclim.org/) with a resolution of 2.5′.

It is necessary to analyze environmental variables before they can be used for niche simulation calculations to prevent the multicollinearity of the variables from causing overfitting of the model, because many bioclimatic variables are spatially related (Graham, 2003). ArcGIS10.2 was employed to examine the relationships among all the environmental variables. If the correlation coefficient of both variables was greater than or equal to 0.8, we removed the relevant environment variables with a lower contribution rate (Pan et al., 2020). A total of 10, 10, and 9 environmental variables based on the MaxEnt model analysis and the Spearman coefficient method were used to predict *S. ciliatum*, *S. nepalense*, and *S. yunnanense* distributions in China, respectively (Table 1).

**TABLE 1:** Environmental variables selected in MaxEnt model

Percentage contribution (%)			
Bioclimatic variables (symbol)	*Satyrium ciliatum*	*Satyrium nepalense*	*Satyrium yunnanense*
Mean diurnal range (bio2)	0.6	0.3	2.5
Isothermality (bio3)	52	61.8	28.6
Temperature seasonality (standard deviation ×100) (bio4)	12.7	14	9.3
Max temperature of warmest month (bio5)	2.6		
Lower temperature of coldest month (bio6)		13.1	54.3
Mean temperature of wettest quarter (bio8)		0	
Mean temperature of coldest quarter (bio11)	11.3		
Precipitation of driest month (bio14)			18
Precipitation seasonality coefficient of variation (bio15)			0.2
Precipitation of warmest quarter (bio18)	9.1	3.8	
Precipitation of coldest quarter (bio19)	1.2	1.7	
Aspect	7.3	3.4	2
Altitude	2.8	1.4	0
Slope	0.4	0.5	1.2

### 2.3 Optimization of model parameters and model building

The feature combination (FC) and regularization multiplier (RM) were adjusted by the ENMeval package in R 4.0.2 software (Yan et al., 2021). There are five FCs in the MaxEnt model: linear, quadratic, product, threshold, and hinge (Phillips et al., 2006). The MaxEnt default parameters are RM = 1, FC = LQHPT. To optimize the model, we set RM to 0.5–4, increased 0.5 each time, and adopted 8 regularization and 6 FCs, namely, L, LQ, H, LQH, LQHP, and LQHP (Li et al., 2020). The ENMeval data package was used to test the 48 parameter combinations and the model's fitting and complexity according to the Delta in Akaike information criterion models delta.AICc and AUC.diff (Zhao et al., 2021).

Data of *Satyrium* species occurrence and each environmental variable were imported into the MaxEnt model, and MaxEnt software (http://www.cs.princeton.edu/, version 3.4.1) was employed for simulation. To validate the model's overall predictive performance, 75% of occurrence records were used for training and the remaining 25% were used for testing (Fielding & Bell, 1997). The background points and the number of iterations were set at less than10,000 and 1000. An average of 10 repetitions was the final output result. In the end, the main environmental variables affecting *Satyrium* species' distribution were evaluated based on the jackknife test results and the contribution rate of environmental variables. Values from the receiver operating characteristic (ROC) and true skill statistic (TSS) were applied to evaluate the model's accuracy (Jiang et al., 2018; Xu et al., 2019). It has been shown that model simulation results are excellent when the values of the area under the curve (AUC) and TSS are >0.9 (Janitza et al., 2013; McIntyre et al., 2017; Phillips et al., 2006).

### 2.4 Classification of suitable habitat

We imported the MaxEnt model outputs into ArcGIS to convert them into raster data using the conversion tool. Additionally, suitable *Satyrium* species habitats were determined by a reclassification tool (Yan et al., 2021) and placed into four groups: highly suitable habitat (0.60–1.00), moderately suitable habitat (0.40–0.60), poorly suitable habitat (0.20–0.40), and unsuitable habitat (0–0.20) (Yang et al., 2013).

### 2.5 Core distributional shifts

Under current and future climate circumstances, the SDM toolbox was used to calculate shifting trends in the area of suitable habitat based on Python, as well as changes in the centroid of the area of suitable habitat (Etherington, 2011). The toolbox is a Python‐based GIS application (Brown et al., 2017). Using the toolbox, the researchers calculated shifting trends in areas of suitable habitats and compared the centroids of the regions between current and future climate conditions. The study provides information about the core shifts and distributions of *S. ciliatum*, *S. nepalense*, and *S. yunnanense*. These species' distributional changes were reduced to a single centroid (central) point, and a vector file was constructed to show the amount and direction of expected change over time. Finally, we examined how the centroid changed with different SDMs to determine whether there were any distribution shifts.

## 3 RESULTS

### 3.1 Model optimization and its accuracy

**FIGURE 2:**
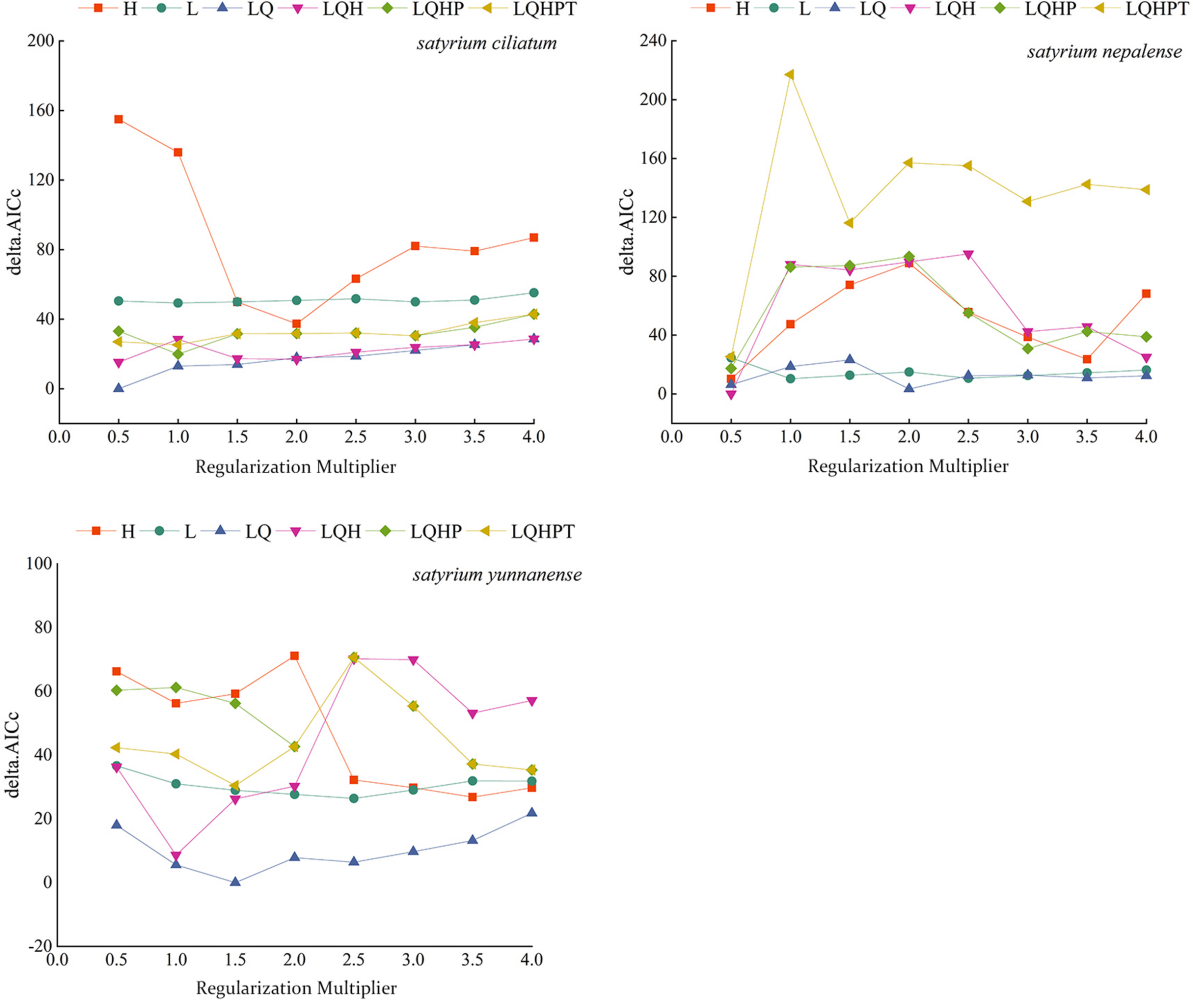
Evaluation metrics of MaxEnt generated by ENMeval

**FIGURE 3:**
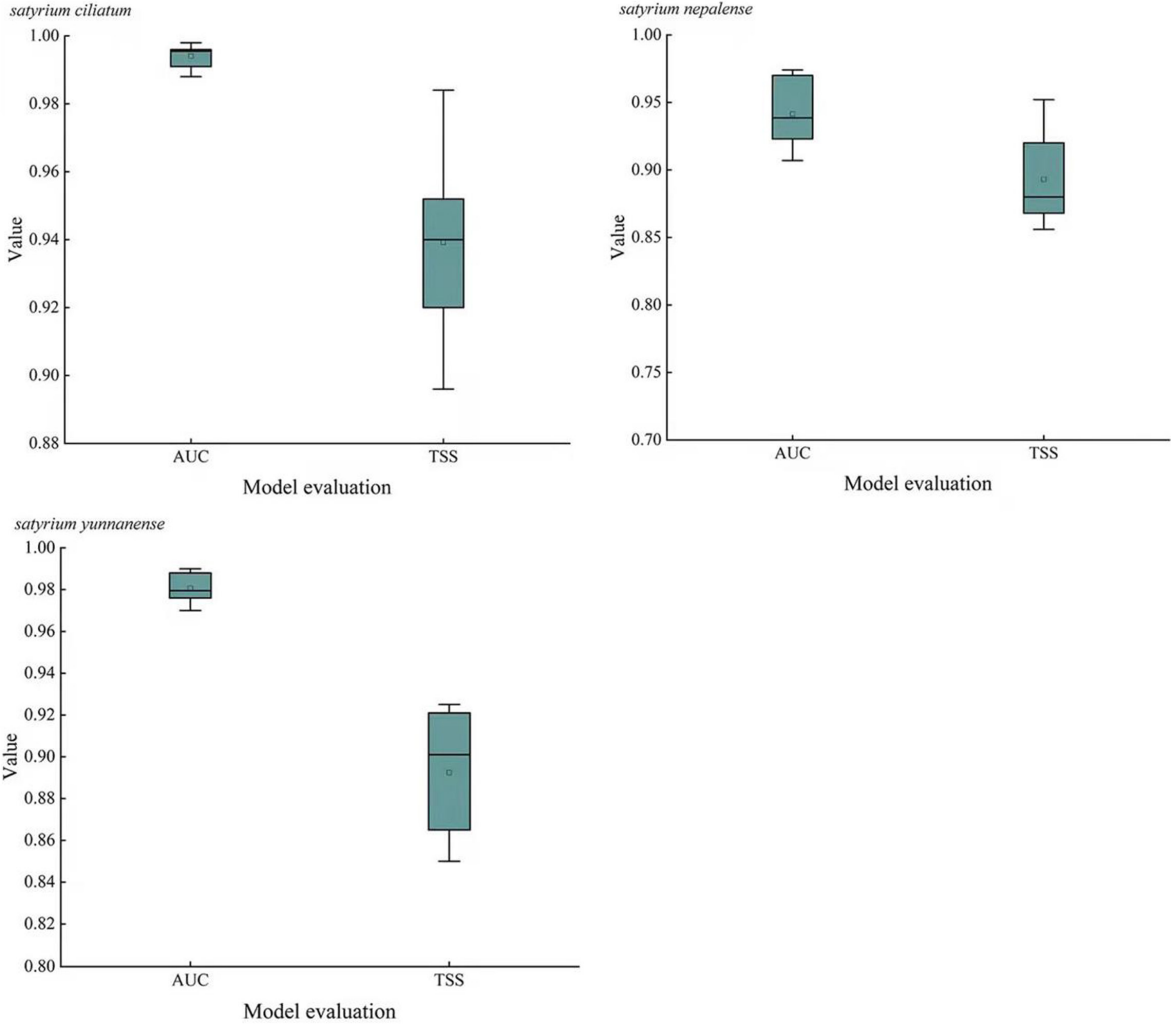
Model evaluation results of AUC value and TSS value

For *S. ciliatum*, under the MaxEnt default parameter, the RM = 1, FC = LQPHT, and delta.AICc = 26.990. When RM = 0.5, FC = LQ, and delta.AICc = 0, the model is optimal (Table S1). Therefore, RM = 0.5 and FC = LQ were selected as the model's final parameters (Figure 2). Under modified parameters, the average values of AUC and TSS indicated an accurate prediction result with values of 0.994 and 0.939 (Figure 3). For *S. nepalense*, under the MaxEnt default parameter, RM = 1, FC = LQPHT, and delta.AICc = 217.062. When RM = 0.5, FC = LQH, delta.AICc = 0, the model is optimal (Table S1). Therefore, RM = 0.5 and FC = LQH were selected as the model's final parameters (Figure 2). Under the modified parameters, the average values of AUC and TSS indicated an accurate prediction result with values of 0.941 and 0.890 (Figure 3). For *S. yunnanense*, under the MaxEnt default parameter, RM = 1, FC = LQPHT, and delta.AICc = 40.356. When RM = 1.5, FC = LQ, and delta.AICc = 0, the model is optimal (Table S1). Therefore, RM = 1.5 and FC = LQ were selected as the final parameters of the model (Figure 2). Under the modified parameters, the average values of AUC and TSS indicated an accurate prediction result with values of 0.981 and 0.894 (Figure 3).

### 3.2 Important environmental variables

The jackknife test results indicated that the top three environmental variables with the largest effect on regularized training gains were as follows: isothermality (bio3), temperature seasonality (bio4), and mean temperature of coldest quarter (bio11) for *S. ciliatum*; isothermality (bio3), temperature seasonality (bio4), and precipitation of the coldest quarter (bio19) for *S. nepalense*; isothermality (bio3), temperature seasonality (bio4), and lower temperature of coldest month (bio6) for *S. yunnanense* when modeling with a single environmental variable (Figure S1). The contribution rate of environmental variables could also be used to evaluate each environment's importance. The contribution rate of each variable was combined with the distribution of *Satyrium* species (Table 1). Isothermality (bio3, 52%) and temperature seasonality (bio4, 12.7%) for *S. ciliatum* reached 64.7%. Isothermality (bio3, 61.8%) and temperature seasonality (bio4, 14%) for *S. nepalense* reached 75.8%, much greater than the other contribution rates. Isothermality (bio3, 28.6%) and lower temperature of coldest month (bio6, 54.3%) for *S. yunnanense* reached 82.9%. The major environmental variables influencing the geographical distribution of *S. ciliatum* were isothermality (bio3), temperature seasonality (bio4), and mean temperature of coldest quarter (bio11). Environmental variables like isothermality (bio3), temperature seasonality (bio4), and precipitation of coldest quarter (bio19) affected the geographical distribution of *S. nepalense*; and environmental variables like isothermality (bio3), temperature seasonality (bio4), and lower temperature of coldest month (bio6) affected the geographical distribution of *S. yunnanense*.

The response curve was used to calculate the thresholds (existence probability >0.6) for the main bioclimatic parameters (Figure S2). For *S. ciliatum*, isothermality (bio3) ranged from 44 to 51.5, temperature seasonality (bio4) ranged from 420 to 580, and mean temperature of coldest quarter (bio11) ranged from 1°C to 13°C. For *S. nepalense*, isothermality (bio3) ranged from 44 to 53, temperature seasonality (bio4) ranged from 400 to 580, and precipitation of coldest quarter (bio19) ranged from 25 to 80 mm. For *S. yunnanense*, isothermality (bio3) ranged from 41 to 53, temperature seasonality (bio4) ranged from 300 to 600, and lower temperature of coldest month (bio6) ranged from −6 to 6°C.

### 3.3 Current potential distributions of *S. ciliatum, S. nepalense,* and *S. yunnanense*

**FIGURE 4:**
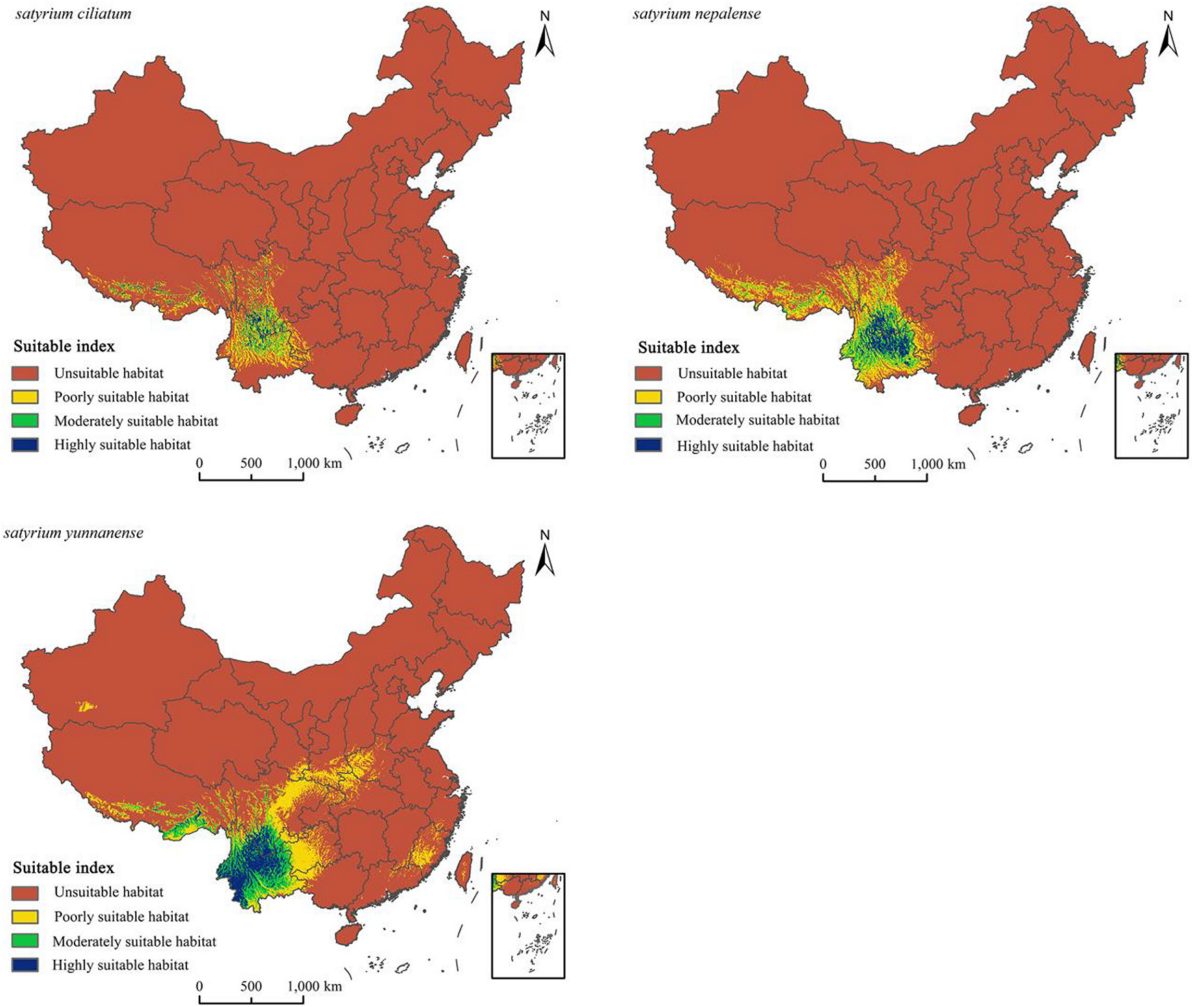
Potential current and suitable habitats for *Satyrium ciliatum*, *Satyrium nepalense*, and *Satyrium yunnanense* in China

As Figure 4 shows, *S. ciliatum* is mainly distributed in Southwest China. Highly suitable habitats are located at the border of Yunnan and Sichuan. Eastern Yunnan and Southern Sichuan have moderately suitable habitats. Poorly suitable habitats cover a broad area, primarily in Northern and Central Yunnan, Southern Tibet, Western Sichuan, and Western Guizhou. There is also a sporadic distribution in Western Guangxi. *S. nepalense* is mainly distributed in Southwest China. Highly suitable habitats are distributed in Central Yunnan. Western and Eastern Yunnan, as well as Southern Sichuan, have moderately suitable habitats. Poorly suitable habitats are located in Southern Yunnan, Western and Southern Sichuan, Southern Tibet, Western Sichuan, and Western Guangxi. *S. yunnanense* is mainly distributed in Southwest China. Highly suitable habitats are distributed in Yunnan. Moderately suitable habitats are located at the border of Yunnan, Guizhou, and Sichuan. Poorly suitable habitats are distributed in Western Guizhou, Central Sichuan, Southern Shaanxi, Western Henan, Southern Fujian, Southern Tibet, and Western Guangxi. The total suitable area for *S. yunnanense* is more than that of *S. nepalense* and *S. ciliatum* according to the mentioned climatic conditions, and these areas account for 9.35%, 6.43%, and 3.39%, respectively, of the total land area of China (960 × 10^4^ km^2^).

### 3.4 Potentially suitable climatic distributions of *S. ciliatum, S. nepalense,* and *S. yunnanense* in the future

**FIGURE 5:**
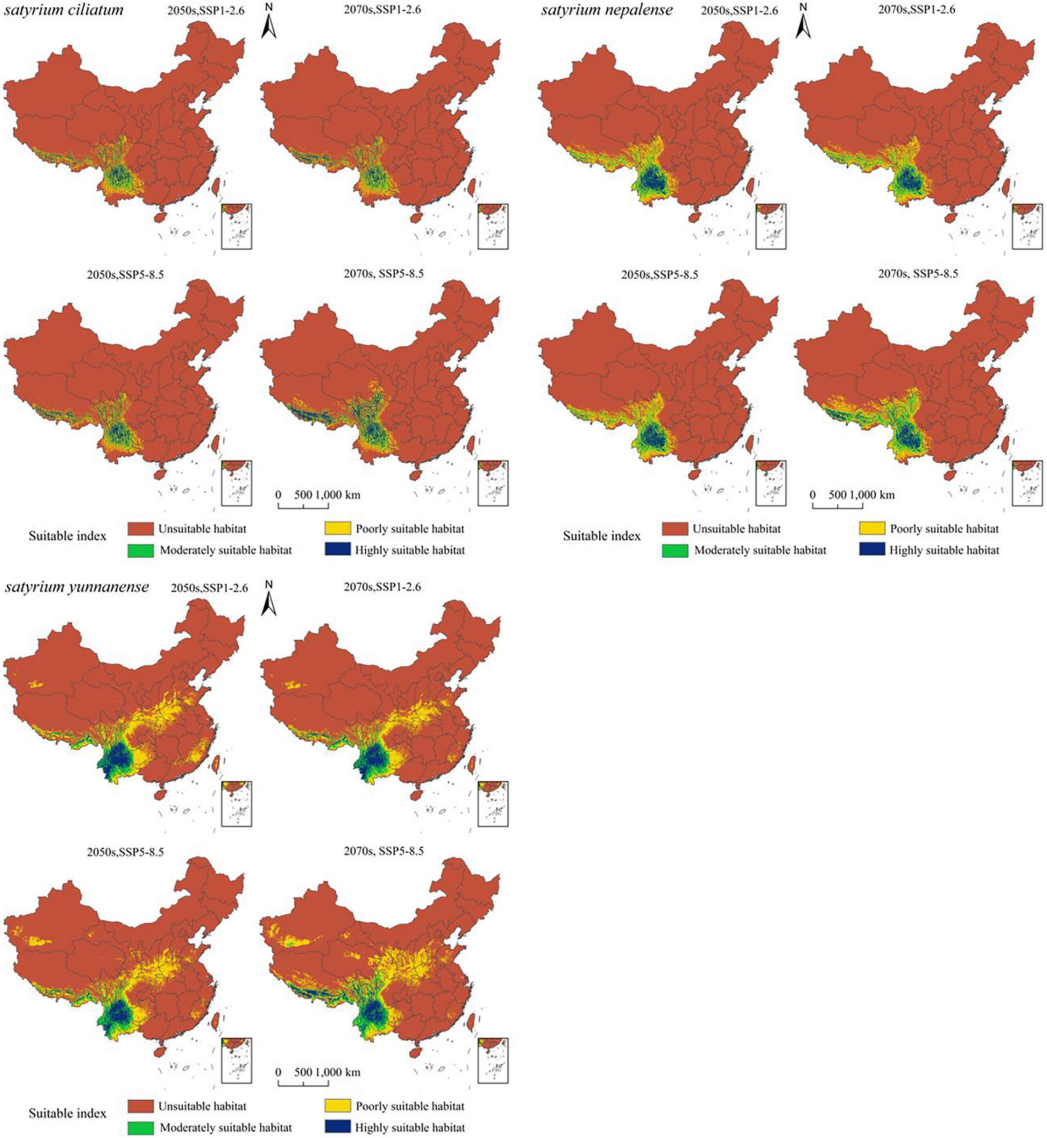
Potential distributions of *Satyrium ciliatum*, *Satyrium nepalense*, and *Satyrium yunnanense* under future climatic scenarios in China

Figure 5 shows the potential distributions of *S. ciliatum*, *S. nepalense*, and *S. yunnanense* in 2050 and 2070 based on two different shared SSPs (SSP1‐2.6 and SSP5‐8.5). A suitable habitat for *S. ciliatum* will be found in parts of Sichuan, parts of Yunnan, Southern Tibet, and the border between Chongqing and Sichuan. The order of total suitable area of different climate models of *S. ciliatum* was as follows: 2070SSP5‐8.5 (69.46 × 10^4^ km^2^) > 2050SSP5‐8.5 (51.83 × 10^4^ km^2^) > 2050SSP1‐2.6 (49.36 × 10^4^ km^2^) > 2070SSP1‐2.6 (48.1 × 10^4^ km^2^) > Current (32.51 × 10^4^ km^2^). The area of suitable habitat in the future was increased compared with the area of highly suitable habitat in current climatic conditions. The order of highly suitable area of different climate models of *S. ciliatum* was as follows: 2070SSP5‐8.5 (21.94 × 10^4^ km^2^) > 2050SSP5‐8.5 (12.19 × 10^4^ km^2^) > 2050SSP1‐2.6 (9.92 × 10^4^ km^2^) > 2070SSP1‐2.6 (9.74 × 10^4^ km^2^) > Current (3.27 × 10^4^ km^2^).

For *S. nepalense*, the suitable habitat is mostly located in parts of Yunnan, parts of Sichuan, Southern Tibet, and along the border between Sichuan and Chongqing. The order of total suitable area of different climate models of *S. nepalense* was as follows: 2070SSP5‐8.5 (97.52 × 10^4^ km^2^) > 2050SSP1‐2.6 (76.42 × 10^4^ km^2^) > 2070SSP1‐2.6 (75.15 × 10^4^ km^2^) > 2050SSP5‐8.5 (74.75 × 10^4^ km^2^) > Current (61.76 × 10^4^ km^2^). The area of suitable habitat in the future was increased compared with the area of highly suitable habitat under current climatic conditions. The order of highly suitable area of different climate models of *S. nepalense* was as follows: 2070SSP5‐8.5 (16.60 × 10^4^ km^2^) > 2050SSP1‐2.6 (13.07 × 10^4^ km^2^) > 2050SSP5‐8.5 (11.95 × 10^4^ km^2^) > 2070SSP1‐2.6 (11.51 × 10^4^ km^2^) > Current (8.76 × 10^4^ km^2^).

For *S. yunnanense*, the suitable habitat is mostly located in Yunnan, the middle of Sichuan, Southern Tibet, and the border between Sichuan and Chongqing. The order of total suitable area of different climate models of *S. yunnanense* was as follows: 2070SSP5‐8.5 (163.3 × 10^4^ km^2^) > 2050SSP5‐8.5 (124.33 × 10^4^ km^2^) > 2050SSP1‐2.6 (123.37 × 10^4^ km^2^) > 2070SSP1‐2.6 (111.33 × 10^4^ km^2^) > Current (89.73 × 10^4^ km^2^). The area of suitable habitat in the future was increased compared with the area of highly suitable habitat under current climatic conditions. The order of highly suitable area of different climate models of *S. yunnanense* was as follows: 2070SSP5‐8.5 (25.12 × 10^4^ km^2^) > 2050SSP1‐2.6 (20.91 × 10^4^ km^2^) > 2070SSP1‐2.6 (19.58 × 10^4^ km^2^) > 2050SSP5‐8.5 (18.25 × 10^4^ km^2^) > Current (16.64 × 10^4^ km^2^).

### 3.5 Future changes in suitable habitat areas in highly suitable centroid distributions

**FIGURE 6:**
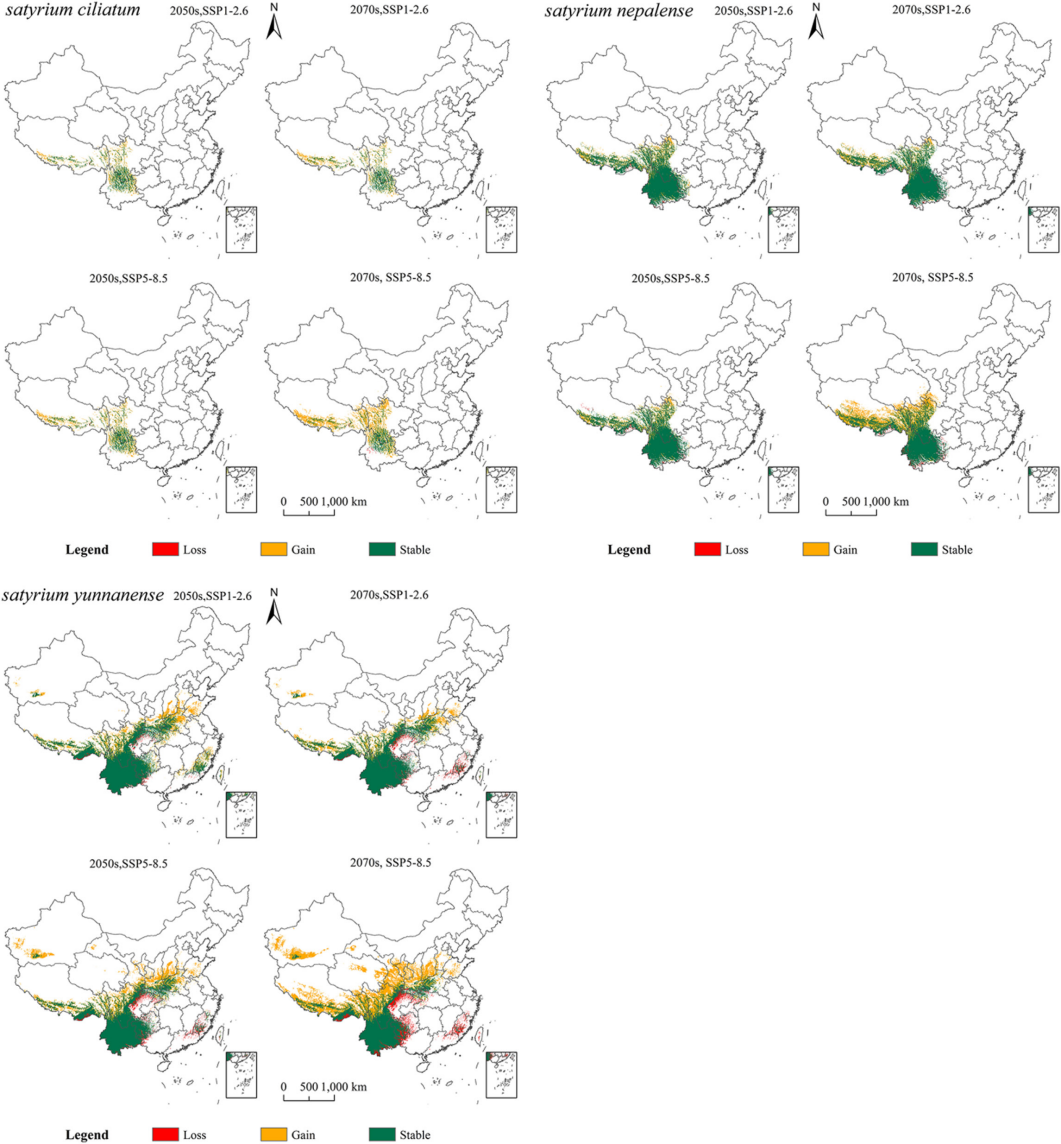
Alterations to possible geographical distributions of *Satyrium ciliatum*, *Satyrium nepalense*, and *Satyrium yunnanense* in climate change scenarios

In regard to future changes in suitable habitat areas, both the gained and lost areas of *Satyrium* will increase in emission concentrations; however, the gained area will be larger than the lost area (Figure 6). According to SSP5‐8.5, the number of increased and decreased areas were the highest compared with the current simulation results. For *S. ciliatum*, by the 2050s, the area of total suitable habitat would increase to 9.65 × 10^4^ km^2^ (SSP1‐2.6) and 12.63 × 10^4^ km^2^ (SSP5‐8.5), and the gained territory would be in Southern Tibet and Northwest Sichuan. By the 2070s, the areas would increase to 9.56 × 10^4^ km^2^ (SSP1‐2.6) and 26.33 × 10^4^ km^2^ (SSP5‐8.5), and the new territory would be in Southern Tibet, Northwest Sichuan, and Northern Yunnan. The decreased habitat would mainly be distributed in Tibet.

For *S. nepalense*, by the 2050s, the area of total suitable habitat would increase to 13.43 × 10^4^ km^2^ (SSP1‐2.6) and 12.42 × 10^4^ km^2^ (SSP5‐8.5), and the gained territory would be in Southern Tibet and Northwest Sichuan. By the 2070s, the areas would increase to 12.70 × 10^4^ km^2^ (SSP1‐2.6) and 36.86 × 10^4^ km^2^ (SSP5‐8.5), and the gained territory would be in Southern Tibet, Northwest Sichuan, and Southwest Gansu. The decreased areas would be fewer, and they would mainly be located in parts of Tibet and Yunnan.

For *S. yunnanense*, by the 2050s, the area of total suitable habitat would increase to 34.15 × 10^4^ km^2^ (SSP1‐2.6) and 41.42 × 10^4^ km^2^ (SSP5‐8.5), and Southern Shaanxi, Southern Shanxi, Northern Henan, and Central Shandong would be the new provinces. By the 2070s, the areas would increase to 26.87 × 10^4^ km^2^ (SSP1‐2.6) and 89.29 × 10^4^ km^2^ (SSP5‐8.5), and Southwestern Xinjiang, Southern Tibet, Northern Sichuan, Southern Shaanxi, Southern Gansu, Northern Henan, and Central Shandong would gain new territory. The decreased areas would mainly be distributed in parts of Guangxi, Fujian, Chongqing, and Yunnan.

### 3.6 Core distributional shifts

**FIGURE 7:**
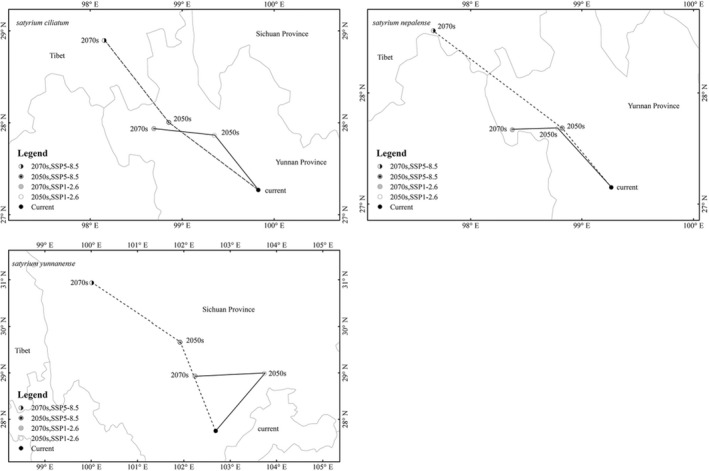
The centroid distributional shifts for *Satyrium ciliatum*, *Satyrium nepalense*, and *Satyrium yunnanense*

Figure 7 shows that the centroid of *S. ciliatum*'s current habitat is located in Central Yunnan Province at 99°84′E and 27°27′N. Under SSP1‐2.6, the centroid of suitable habitat will shift to 99°35′E and 27°87′N in the 2050s, and 98°69′E and 27°94′N in the 2070s. Under SSP5‐8.5, the centroid of the future suitable region will be located at 98°86′E and 28°01′N in the 2050s. Under SSP5‐8.5, the suitable area's centroid will relocate to 98°15′E and 28°91′N in the 2070s. In summary, we can see that the core distribution of *S. ciliatum* will shift to the northwest under both future emission trajectories (SSP1‐2.6, SSP5‐8.5). The centroid of the existing habitat for *S. nepalense* is located in Southern Yunnan Province at 99°28′E and 27°15′N. Under SSP1‐2.6, the centroid of the suitable region will shift to 98°79′E and 27°69′N in the 2050s and 98°38′E and 27°69′N in the 2070s. Under SSP5‐8.5, the centroid of the future suitable region will be located at 98°82′E and 27°69′N in the 2050s. The centroid of the suitable region will relocate to 97°67′E and 28°56′N in the 2070s under SSP5‐8.5. The centroid of the current habitat for *S. yunnanense* is located in south Sichuan province at 102°67′E and 27°75′N. Under SSP1‐2.6, the centroid of the suitable area will shift to 103°73′E and 29°02′N in the 2050s, and 102°22′E and 28°94′N in the 2070s. According to SSP5‐8.5, the suitable area's centroid will relocate to 101°91′E and 29°68′N by the 2050s, then to 100°05′E and 30°94′N in the 2070s.

## 4 DISCUSSION

The *Satyrium* plant species are important resources, but they are endangered and rare (Mishra & Saklani, 2012). MaxEnt models were applied to predict current and future potential distribution areas of *S. ciliatum*, *S. nepalense*, and *S. yunnanense*. According to the four different future scenarios, the suitable habitat area for *Satyrium* in China will be extended. These results are consistent with the results of a study on the *Houttuynia cordata* Thunb (Ceercao) habitat suitability; the potential suitable area in the high‐emission scenario was greater than that in the low‐emission scenario in the study (Liu et al., 2021). The increased precipitation in China in the SSP5‐8.5 emission scenario was higher than in the low concentration emission scenario (Yue et al., 2021). This shows the increased precipitation in the high concentration emission scenario can solve the limitations of precipitation on distribution of species, and the increased precipitation in the low concentration emission scenario cannot reduce or solve the limitations of precipitation on the distribution of species. This may also be the reason the most suitable habitat area and the increasing area of the *Satyrium* species are the largest in the high concentration emission scenario, particularly the SSP5‐8.5 scenario. The prediction shows that the upcoming future will be the most suitable for the growth of the *Satyrium* species. It shows that, generally, the suitable habitat is stable and the species will not face extinction due to climate change. This study shows the new areas for species distribution. The findings are in agreement with those of previous studies, indicating that in some regions, habitat suitability of plant species improves under climate change conditions (Feng et al., 2020; Li et al., 2019). The distribution of species under future climatic scenarios predicts that the area of species covering 20% of the earth's surface will face the risk of extinction, and about 15%–37% of the species will be endangered (Thomas et al., 2004). One of the reasons for the endangered status of *Satyrium* is that most *Satyrium* species are hermaphroditic. Some species rely on birds and other animals for long‐distance transmission, the species population is small, and the quality of seed development is low because of long‐distance pollination (Johnson et al., 2011). The suitable habitat area for *Satyrium* in China will move to higher altitude areas in the northwest side of China in the 2050s and 2070s, where the humidity and habitat are suitable for the growth of the *Satyrium* species. The centroid of the distribution of endangered plant *Semiliquidambar cathayensis* (Hamamelidaceae) will move northward (Ye et al., 2020). The distribution of suitable habitat of *Paeonia delavayi* will shift to higher elevations (Zhang et al., 2018). With the global warming, more plants tend to migrate to high altitude and high latitude areas (Sekercioglu et al., 2008). This study's results provide a reliable basis for protecting *Satyrium*. People should effectively protect its native habitat and population. Nature reserves for *Satyrium* should be set up now according to the prediction results, and they should be strengthened to minimize human‐made damage.

The results show that isothermality (bio3) and temperature seasonality (bio4) are the common factors in the three species, indicating that the main environmental variable affecting the appropriate distribution of *Satyrium* is temperature. Zhao et al. (2021) used the MaxEnt model to predict the potential suitable habitat of Chinese fir in China. Their results showed that temperature factors are more important than precipitation factors influencing habitat suitability for Chinese fir. Lou et al. (2015) used regression models to predict tea crop yield responses to climate change, finding that temperature plays a significant role in tea production and quality. Environmental variables like temperature can estimate plant distribution patterns (Deblauwe et al., 2016; Wang et al., 2021). In regard to *S. nepalense*, precipitation is one of the environment variables influencing its geographic distribution. *Satyrium* is a perennial terrestrial orchid distributed in Southwest China (Deng et al., 2019). Terrestrial orchids are typically cold tolerant, live in low‐temperature environments, and are highly distributed in areas with large amounts of annual rainfall (Phillips et al., 2011; Poff et al., 2016). More studies have shown that the dominant variables restricting geographical distribution of plants are energy supply, plenty of water, and cold tolerance (Zhou & Wang, 2000). Based on the prediction of the MaxEnt model, the area of the current potential suitable habitat of *Satyrium* is mainly located in Southwest China, which has a typical subtropical monsoon climate (Luo et al., 2021). Previous studies have indicated that precipitation is the main variable affecting plant growth, regeneration, nutrient cycles, and community productivity in different habitats (Miranda et al., 2011). Climate change can determine geographical distribution of species, and geographical distribution of species can respond to changes in climate (Warren et al., 2021). Global warming has changed the structures of terrestrial ecosystems, which have changed the habitats and geographical distribution functions of species in turn (Ghini et al., 2012). For instance, climate, topography, soil, human disturbance, and spatial constraints are significant to the distribution of many different spatial scales (Eiserhardt et al., 2011; Parisien & Moritz, 2009). The environmental data used in the study included bioclimatic variables and topographic variables in WorldClim. We did not study soil type, land use, human activities, biological interaction, and other factors that influence the distribution of *Satyrium*. The more factors that are included, the more accurate the prediction will be. Therefore, other factors affecting the potential suitable habitat of *Satyrium* should be studied in the future, with different niche prediction models predicting areas of potential suitable habitats. This will make the prediction of results more reliable.

Generally, the evaluation index of prediction model accuracy is the ROC curve, which has the following advantage: the AUC value is not affected by the threshold and can be used for the comparison of various models (Fielding & Bell, 1997). However, further research shows that it is inaccurate to evaluate accuracy only by AUC value because it cannot reflect wrong spatial distribution information, and calculate the omission rate and error rate by a single method (Lobo et al., 2008; Peterson et al., 2008). Nowadays, the information measurement index AUC is used to select the ideal setting parameters of MaxEnt (Muscarella et al., 2014); the method of combining omission rate and AUC value is used to obtain the best parameters (Radosavljevic & Anderson, 2014); TSS value is used to evaluate accuracy (Bedia et al., 2011); and multiple parameters are set to select the optimal result (Elith et al., 2010; Warren & Seifert, 2011). TSS can accurately correct the overall accuracy of the model without being affected by the size of the species validation data set and without depending on the model threshold, therefore, AUC and TSS values are used as evaluation indexes in this prediction (Bedia et al., 2011).

## 5 CONCLUSION

In the study, the potential suitable *Satyrium* habitat in China was predicted based on the MaxEnt model optimized by the ENMeval package. Temperature was the common variable in the three species. In regard to *S. nepalense*, precipitation is one of environment variables influencing its geographic distribution. The potential geographical distribution of *Satyrium* under current climatic conditions is primarily on the southwest side of China. *Satyrium*'s highly suitable habitat area, moderately suitable habitat area, and total suitable habitat area were predicted to increase under future climate change scenarios. The highly suitable habitat area of *Satyrium* showed a tendency to shift to areas with higher elevation. In regard to future changes in suitable habitat areas, both the gained and lost areas of *Satyrium* will increase in emission concentrations; however, the gained area will be larger than the lost area. These results can provide theoretical guidance for protecting endangered plants in China and offer further reference for biodiversity protection in China.

## AUTHOR CONTRIBUTIONS

**Xianheng Ouyang:** Conceptualization (lead); data curation (lead); resources (lead); software (lead); visualization (lead); writing – original draft (lead); writing – review and editing (lead). **Shihao Bai:** Investigation (lead); methodology (lead); writing – review and editing (equal). **Garry Brien Strachan:** Writing – review and editing (supporting). **Anliang Chen:** Funding acquisition (lead); project administration (lead); resources (lead); writing – review and editing (equal).

## CONFLICT OF INTEREST

The authors declare that they have no conflict of interest for the publication of this study.

### OPEN RESEARCH BADGES

This article has earned Open Data, Open Materials and Preregistered Research Design badges. Data, materials and the preregistered design and analysis plan are available at Dryad Digital Repository (https://doi.org/10.5061/dryad.k98sf7m6v).

## Supporting information


Supplementary Material

## Data Availability

All authors agreed to deposit data from this manuscript in a public repository. Data were submitted to Dryad (the DOI number is https://doi.org/10.5061/dryad.k98sf7m6v).
