# Supplementary material for: Simulation of the potential distribution of rare and endangered Satyrium species in China under climate change
Source: Ecol Evol. 2022 Jul 11;12(7):e9054. doi: 10.1002/ece3.9054 (PMC9273742; doi:10.1002/ece3.9054)
Supplement: Supplementary file 1 — Supplementary Material [file ECE3-12-e9054-s001.docx]

**APPENDIX**

**TABLE S1. Evaluation results of MaxEnt model under different parameter setting of *Satyrium ciliatum*, *Satyrium nepalense*, and *Satyrium yunnanense.***

| ***Satyrium ciliatum*** | | | | | |
| --- | --- | --- | --- | --- | --- |
| Setting | FC | RM | AUC.diff | AICc | delta.AICc |
| Default | LQPHT | 1 | 0.014 | 949.537 | 26.990 |
| Optimized | LQ | 0.5 | 0.015 | 922.547 | 0 |
| ***Satyrium nepalense*** | | | | | |
| Setting | FC | RM | AUC.diff | AICc | delta.AICc |
| Default | LQPHT | 1 | 0.058 | 653.751 | 217.062 |
| Optimized | LQH | 0.5 | 0.043 | 436.689 | 0 |
| ***Satyrium yunnanense*** | | | | | |
| Setting | FC | RM | AUC.diff | AICc | delta.AICc |
| Default | LQPHT | 1 | 0.0143 | 398.777 | 40.356 |
| Optimized | LQ | 1.5 | 0.0151 | 358.421 | 0 |
